# Kaempferol Inhibits the Primary Attachment Phase of Biofilm Formation in *Staphylococcus aureus*

**DOI:** 10.3389/fmicb.2017.02263

**Published:** 2017-11-15

**Authors:** Di Ming, Dacheng Wang, Fengjiao Cao, Hua Xiang, Dan Mu, Junjie Cao, Bangbang Li, Ling Zhong, Xiaoyun Dong, Xiaobo Zhong, Lin Wang, Tiedong Wang

**Affiliations:** ^1^College of Animal Science, Jilin University, Changchun, China; ^2^College of Animal Science and Technology, Jilin Agricultural University, Changchun, China; ^3^Department of Pharmacology, College of Basic Medical Science, Jilin University, Changchun, China; ^4^Key Laboratory of Zoonosis Research, Ministry of Education, Institute of Zoonosis, College of Veterinary Medicine, Jilin University, Changchun, China

**Keywords:** *Staphylococcus aureus*, biofilm, kaempferol, inhibitor, primary, adhesion

## Abstract

The ability to form biofilms on surfaces makes *Staphylococcus aureus* the main pathogenic factor in implanted medical device infections. The aim of this study was to discover a biofilm inhibitor distinct from the antibiotics used to prevent infections resulting from *S. aureus* biofilms. Here, we describe kaempferol, a small molecule with anti-biofilm activity that specifically inhibited the formation of *S. aureus* biofilms. Crystal violet (CV) staining and fluorescence microscopy clearly showed that 64 μg/ml kaempferol inhibited biofilm formation by 80%. Meanwhile, the minimum inhibitory concentration (MIC) and growth curve results indicated that kaempferol had no antibacterial activity against the tested bacterial strain. Kaempferol inhibited the primary attachment phase of biofilm formation, as determined by a fibrinogen-binding assay. Moreover, a fluorescence resonance energy transfer (FRET) assay and quantitative real-time reverse transcription polymerase chain reaction (qRT-PCR) analyses revealed that kaempferol reduced the activity of *S. aureus* sortaseA (SrtA) and the expression of adhesion-related genes. Based on these results, kaempferol provides a starting point for the development of novel anti-biofilm drugs, which may decrease the risk of bacterial drug resistance, to prevent *S. aureus* biofilm-related infections.

## Introduction

*Staphylococcus aureus* (*S. aureus*) is an important Gram-positive pathogen that can cause both human and animal diseases (Mistry et al., 2016). Statistically, 20–25% of the population serve as long-term *S. aureus* hosts, whereas 75–80% of the population are intermittent hosts (Harmsen et al., 2010; Singhal et al., 2011). *S. aureus* can cause suppurative inflammation in the dermal mucous membranes and in many other tissues and organs (Lowy, 1998).

Bacterial pathogens with the ability to form biofilms, such as *S. aureus*, easily colonize the surfaces of certain indwelling medical devices (O'Gara and Humphreys, 2001; Von Eiff et al., 2002). Biofilms allow embedded bacteria to resist antimicrobial therapy (Hoyle and Costerton, 1991; Aaron et al., 2002; Parsek and Singh, 2003; Anderson and O'Toole, 2008), for example, by reducing contact with antimicrobial compounds (Aendekerk et al., 2005; Bjarnsholt et al., 2005) or reducing metabolic activity, which lowers the sensitivity to multiple antibiotics (Anderson and O'Toole, 2008). Consequently, the control of biofilm-forming *S. aureus* is very difficult (Costerton et al., 1999; Mah and O'Toole, 2001). The efficacy of single antibiotics against *S. aureus* biofilms in clinical practice is poor (Rogers et al., 2010), and as a result, new therapeutic strategies and molecular data on potential means of disturbing biofilm development are in great demand (Romling and Balsalobre, 2012).

The development of a bacterial biofilm can be divided into the following phases: initial adhesion, proliferation, maturation and diffusion (Otto, 2008; Boles and Horswill, 2011). Three principle strategies target the different stages of biofilm development: attachment inhibition, biofilm architecture disruption and signal transduction interference (Chung and Toh, 2014). Various inhibitors such as plant-derived natural compounds or synthesized small molecules (Brackman and Coenye, 2015; Mogosanu et al., 2015), enzymes targeting the matrix (Itoh et al., 2005), an enzyme from an *S. aureus* bacteriophage that degrades the cell wall (Kelly et al., 2012), nanoparticles and silver ions (Jia et al., 2017), polysaccharides and synthetic peptides with anti-biofilm activity (Rendueles et al., 2013; Pletzer and Hancock, 2016) have been discovered.

Plant secondary metabolites are main sources of antimicrobial agents and other pharmaceuticals (Li and Vederas, 2009; Lee et al., 2016). Some biofilm inhibitors derived from plants have been found to exhibit activity against *S. aureus* biofilms, including magnolol (Wang et al., 2011), ellagic acid (Quave et al., 2012), tannic acid (Payne et al., 2013), quercetin (Lee et al., 2013), ginkgolic acids (Lee et al., 2014), eugenol (Yadav et al., 2015), and flavonoids (Cho et al., 2015).

**Figure 1:**
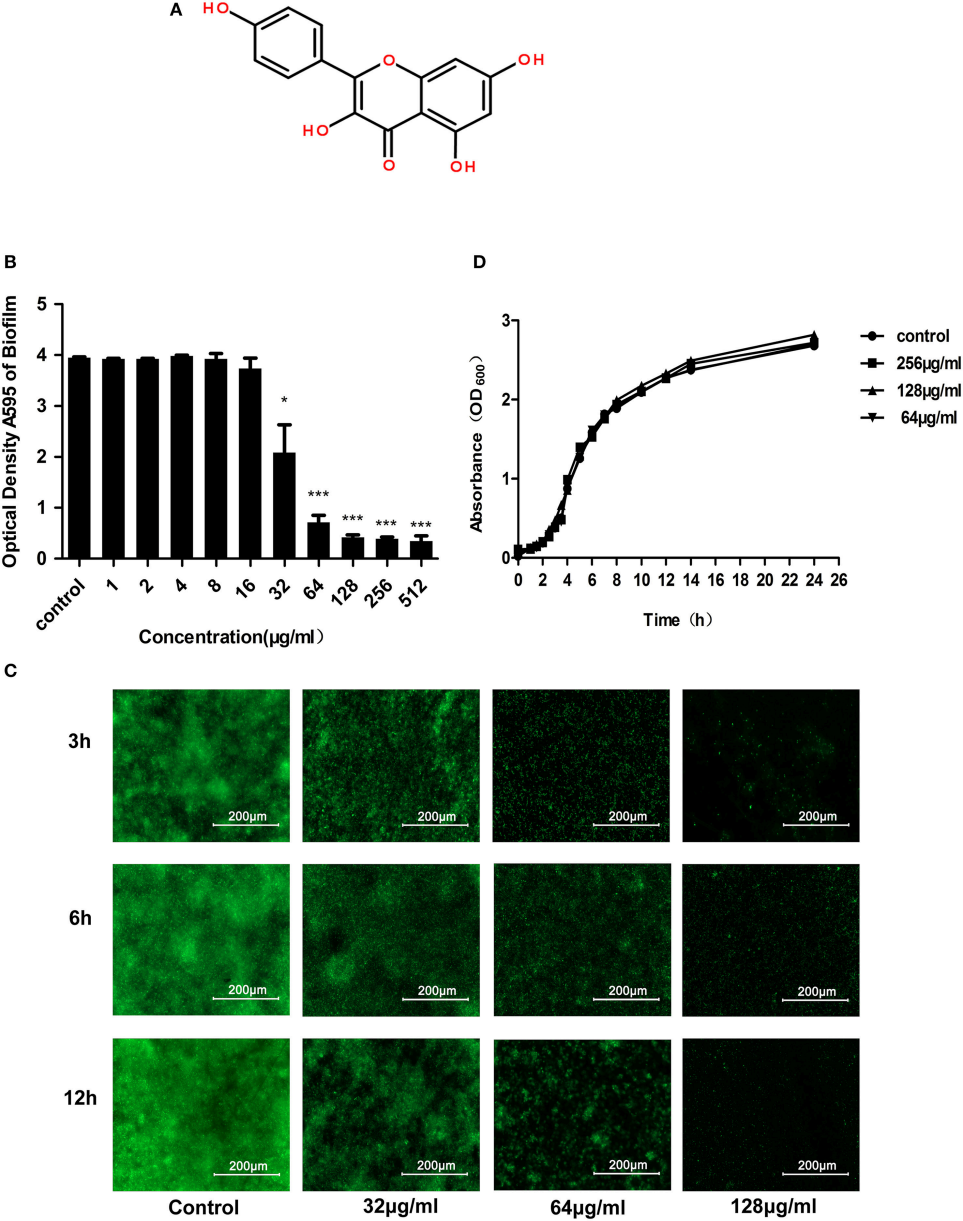
Anti-biofilm and antibacterial activities of kaempferol against *S. aureus* ATCC® 29213™. **(A)** Chemical structure of kaempferol. **(B)** *S. aureus* was incubated with various concentrations of kaempferol for 12 h. CV was used to stain the biofilms. The bound CV was released from the stained cells with 95% ethanol, and the OD595 was measured. Data are represented as the mean ± standard deviation. ^*^P < 0.05, and ^***^P < 0.001 compared to the control. **(C)** Fluorescence microscopy. Scale bars represent 200 μm. **(D)** Growth curve of *S. aureus* ATCC® 29213™ with or without kaempferol. Kaempferol at 64, 128, and 256 μg/ml had no effect on bacterial growth compared with that of the control group.

Despite their great potential for treating biofilm-related infections, the mechanism of action of these agents remains unclear. Our research currently aims to discover small molecule compounds that specifically act on the key virulence factors in bacteria, such as listeriolysin O of *Listeria monocytogenes* and α-hemolysin (Hla) and SrtA of *S. aureus* (Qiu et al., 2012; Wang J. et al., 2015; Wang L. et al., 2015). The ability to form biofilms is now believed to be an important virulence characteristic for some *Staphylococcus* bacteria. Therefore, we screened *S. aureus* biofilm inhibitors from 200 natural compounds preserved in our laboratory and found that kaempferol (Figure 1A) exhibited good anti-biofilm activity.

Kaempferol is a typical flavonol with many biological and pharmacological activities, including antitumor, anti-inflammatory, and antioxidative effects (Ross and Kasum, 2002). In addition, kaempferol is known to inhibit the activity of *S. aureus* PriA helicase (SaPriA) and the activity of bacterial efflux pumps, thereby blocking the growth and survival of antibiotic-resistant *S. aureus* and increasing antimicrobial effectiveness (Brown et al., 2015; Huang et al., 2015). In our study, we tested the capacity of kaempferol to inhibit *S. aureus* ATCC®29213™ biofilm formation and explored the specific stages and potential molecular mechanisms of this activity in depth.

## Materials and methods

### Strains and growth conditions

The *S. aureus* strain ATCC® 29213™ (National Center For Medical Culture Collections) was used in this study for its high ability to form biofilms *in vitro* (Abouelhassan et al., 2014). The ΔSrtA strain was constructed using a method described previously (Chen et al., 2014). The SrtA_ΔN59_ protein was constructed in the previous study by our team (Wang L. et al., 2015). The fluorescent peptide Dabcyl-QALPETGEE-Edans was synthesized by GL Biochem (Shanghai, China). The *S. aureus* ATCC® 29213™ and ΔSrtA strains were cultured in brain–heart infusion (BHI) solution (Oxoid, Basingstoke, UK) supplemented with 0.5% glucose and 3% NaCl at 37°C with shaking at 220 rpm.

### Antimicrobial agents

Kaempferol was purchased from the Chengdu Ruifensi Biotech Company (Chengdu, China) and dissolved in dimethyl sulfoxide (DMSO) (Changchun Baotaike Biotech Company, China) to a storage concentration of 100 mg/ml.

### Biofilm inhibitor screening

The *S. aureus* ATCC® 29213™ strain was cultured overnight and then diluted 1:100 in sterile BHI broth supplemented with 0.5% glucose and 3% NaCl. Then, 200 μl of diluted bacteria was placed in a polystyrene Costar 96-well plate (Tiangen, Changchun Baotaike Biotech Company, China) coated with plasma (20% rabbit freeze-dried plasma, incubated overnight at 4°C). Compounds were added to the assay plates at a final concentration of 512 μg/ml, and the plate was incubated at 37°C for 12 h without shaking. Then, the supernatant was completely removed. After rinsing in phosphate buffered saline (PBS), the biofilms were stained with 12.5 g/l crystal violet (CV) for 10 min, washed again with PBS and dried. Images were obtained using an AMT 2 k charge-coupled device (CCD) camera. Finally, 0.2 ml of ethanol (95%) was added to the CV-stained wells, which were then incubated for 30 min to quantify biofilm growth, and 100 μl of each final dissolved CV solution was transferred into new 96-well plates and measured at an OD of 595 nm using a microplate reader (Infinite® F500, Tecan, Shanghai, China). Each data point consisted of three independent samples. The compounds that showed 90% inhibition compared with the negative control (no compound added) were selected as validated hits.

### MIC and growth curves

The minimum inhibitory concentration (MIC) of kaempferol were determined in accordance with the Clinical and Laboratory Standards Institute (CLSI) Approved Standard M7-A8, (CLSI, Wayne, PA, 2009, pp. 19–91). To obtain growth curves, *S. aureus* was cultured overnight and diluted 1:100 into sterile BHI (supplemented with 0.5% glucose and 3% NaCl) broth with or without kaempferol at different concentrations. The absorbance at 600 nm was measured at different time intervals.

### Fluorescence microscopy

Sytox green was obtained from Invitrogen-Molecular Probes (Oregon). After treatment with 128, 64, and 32 μg/ml kaempferol for 3, 6 and 12 h, the biofilms produced by each group were washed with PBS. To permeabilize the bacterial membranes, the cell growth substrates were soaked in 0.1% (vol/vol) Triton X-100 (VWR) in PBS (PBST) for 15 min. The cells were labeled by replacing the PBST with 0.5 μM Sytox green nucleic acid stain in PBST for 30 min. The cells were then washed with PBS to remove the excess stains. Afterwards, fluorescent images were obtained using a fluorescence microscope (Leica DMRX).

### Fibrinogen-binding assay

The *S. aureus* ATCC® 29213™ strain was cultured overnight and then diluted 1:100 in sterile BHI (supplemented with 0.5% glucose and 3% NaCl) broth containing different concentrations of kaempferol and cultured with shaking at 37°C. All cells were collected by centrifugation (5,000 × g for 5 min) when the OD600 reached 0.5. The cells were suspended in PBS to obtain an OD600 of 1.0. The resuspended cells were placed to polystyrene Costar 96-well plates coated with fibrinogen (incubated overnight with 20 μg/ml bovine fibrinogen at 4°C) and incubated for 1 h at 37°C. The supernatant was removed, and the cells were washed with PBS and fixed with 25% (v/v) formaldehyde. After 30 min, the adherent bacteria were washed again with PBS and stained with 12.5 g/l CV for 10 min. The wells were then washed with PBS and dried. Subsequently, different samples were measured at 595 nm. The percentage of the tested group compared to the control group was used to report results. To ensure reproducibility, each experiment was repeated at least three times.

### Inhibition of sortase A activity

The influence of kaempferol on SrtA was examined by a fluorescence resonance energy transfer (FRET) method (Ton-That et al., 1999; Mazmanian et al., 2002). Sortase A (SrtA), an enzyme that anchors surface proteins to the cell wall of Gram-positive bacteria, cleaves sorting signals between the threonine (T) and glycine (G) of the LPXTG motif (Ton-That et al., 1999). During SrtA catalysis, the change in fluorescence was monitored using the fluorescent peptide substrate Dabcyl-QALPETGEE-Edans of SrtA. The inhibitory effect of kaempferol on the activity of SrtA was measured based on the fluorescence changes. The sortase A (SrtA) activity inhibition assay was conducted according to a method described previously (Bi et al., 2016). The experiment was performed in black 96-well plates. The reaction consisted of 4 μM SrtA_Δ*N*59_ protein, 10 μM peptide substrate and the assay buffer (150 mM NaCl, 5 mM CaCl_2_, 0.1% Triton X-100 and 50 mM Tris-HCl, pH 7.5) in a final volume of 300 μl. The SrtA_Δ*N*59_ protein and different concentrations of kaempferol were added to the plate and incubated at 37°C for 1 h. Then, the peptide substrate was added, and the reaction continued for another 1 h at 37°C. The change in fluorescence intensity was detected using a microplate reader (Infinite®F500, Tecan, Shanghai, China) with 495 nm as the emission wavelength and 350 nm as the excitation wavelength. To ensure the reproducibility of this assay, each reaction was repeated three times.

### Transmission electron microscopy

Transmission electron microscopy (TEM) was performed using a JEM-2100 transmission microscope (JEOL, Tokyo, Japan). Biofilm samples were prepared according to the method described above. For the proteinase K group, proteinase K (2 μg/ml) was added to the pre-established biofilms and incubated for 2 h at 37°C. Samples prepared for TEM imaging were spotted onto formvar-coated copper grids, incubated for 5 min, washed with sterile distilled H_2_O, and negatively stained with 2% uranyl acetate for 60 s (Chu et al., 2016).

### Confocal microscopy

The confocal microscopy experiment was performed using confocal laser-scanning microscopy (Olympus, Shanghai, China). Syto 63 and fluorescein isothiocyanate (FITC) were purchased from Invitrogen Molecular Probes (Oregon). The biofilms were cultured according to the above method on glass cover slides with or without kaempferol (64 μg/ml). The biofilms produced by each group were washed with PBS after 12 h. Syto 63 (100 μM) was added to each well, and the plate was incubated with shaking for 5 min. FITC (0.001%) was then added, and the plate was incubated for another 30 min without shaking. The cells were washed with PBS to remove excess stain. Confocal microscopy images were obtained from NIS-Elements C version 3.2 (Nikon Eclipse).

### RNA isolation

For quantitative real-time reverse transcription polymerase chain reaction (qRT-PCR) experiments, RNA from *S. aureus* cells was isolated using the following procedure. The *S. aureus* ATCC® 29213™ strain was cultured overnight and diluted 1:100 into sterile BHI broth supplemented with 0.5% glucose and 3% NaCl. After culturing at 37°C for 3 h with shaking at 220 rpm, kaempferol was added to a concentration of 64 μg/ml—at which it showed significant anti-biofilm activity—and the culture was incubated again for 5 h. Subsequently, RNA was isolated using the TRIzol (Tiangen, Changchun Baotaike Biotech Company, China) RNA extraction method. The concentration of RNA was assessed using a NanoVue Plus (Biochrom Ltd., Cambridge, UK).

### qRT-PCR

qRT-PCR was used to assess the transcription levels of adhesion-related genes (*fnbA, fnbB, clfA, clfB, sarA*) in *S. aureus* ATCC® 29213™. Gene-specific primers (listed in Table 1) were used for these genes, and appropriate primers were used for 16S rRNA as a housekeeping control to normalize the expression of genes of interest. The isolated RNAs were reverse-transcribed into cDNA using the TransScript® All-in-One First-Strand cDNA Synthesis SuperMix (Quanshijin, Changchun Weierkete Biotech Company, China). Then, qRT-PCR was performed using the TransStart Top Green qPCR SuperMix (Quanshijin, Changchun Weierkete Biotech Company, China) under the following conditions: initial denaturation at 95°C for 10 min followed by 40 cycles of 95°C for 15 s, 60°C for 10 s and 72°C for 10 s. As a negative control, qRT-PCR was performed without cDNA. The experiments were performed three times in parallel, and the data were analyzed using a previously described relative quantitative (2^−ΔΔCt^) method (Livak and Schmittgen, 2001).

**Table 1:** Oligonucleotide primers used in this study.

**Primer name**	**Oligonucleotide (5′−3′)**
*16S rRNA-F*	GCTGCCCTTTGTATTGTC
*16S rRNA-R*	AGATGTTGGGTTAAGTCC
*fnbA-F*	GACCCGCTTCACTAT
*fnbA-R*	ACACCGCTTGACATT
*fnbB-F*	AATAAGGATAGTATGGGTAG
*fnbB-R*	CACAAGTAATGGTCGGT
*clfA-F*	TTGATTGGCGATACG
*clfA-R*	TGACCCTGAAAATGTTA
*clfB-F*	ACGAATGGCGATGTT
*clfB-R*	CACTACGACGACCATA
*sarA-F*	ATGATTGCTATGAGTT
*sarA-R*	TGTTCGCTGATGTATG

#### Statistical analysis

Statistical analyses were conducted using Student's *t*-test with SPSS 13.0 software. The data were expressed as the mean ± standard deviation. Values of *p* < 0.05 were considered statistically significant.

## Results

### Kaempferol inhibits the formation of *S. aureus* biofilms without affecting the growth of planktonic bacteria

According to the screening assay, kaempferol at 512 μg/ml showed 90% inhibition on the biofilm formation representing that it had obvious anti-biofilm activity. To detect the influence of different concentrations of kaempferol on *S. aureus* biofilms, *S. aureus* was co-cultured with kaempferol (1–512 μg/ml) using the microdilution method, which was similar to the MIC assay for planktonic cells in 96-well plates coated with 20% rabbit freeze-dried plasma at 37°C for 12 h without shaking. After 12 h, we stained the biofilm with crystal violet and measured the absorbance at 595 nm. We observed that kaempferol was effective at lower concentrations, and it could inhibit biofilm formation by 80% at 64 μg/ml (Figure 1B).

The nucleic acid dye Sytox Green is a fluorescent indicator that can efficiently label the entire cytoplasm (Hochbaum et al., 2011). Therefore, the effect of kaempferol on biofilms was observed by the fluorescence microscopy. Fluorescent images indicated that biofilm of the control group increased gradually with time, whereas kaempferol dose-dependently inhibited the biofilm formation (Figure 1C).

To test whether the effect of kaempferol on biofilms was dependent on the inhibition of the growth of planktonic bacteria, MIC of kaempferol was determined by MIC experiment. The results showed that the MIC of kaempferol on *S. aureus* was greater than 1,024 μg/ml. In addition, the results of the growth curve were consistent with the MIC results: bacteria treated with different concentrations of kaempferol (64–256 μg/ml) showed the same growth trend as the control group (Figure 1D). These results confirmed that kaempferol did not affect the growth of *S. aureus* at concentrations of 64–256 μg/ml but could significantly inhibit the formation of *S. aureus* biofilms.

### Kaempferol specifically inhibits the initial attachment phase of *S. aureus* biofilm formation

**Figure 2:**
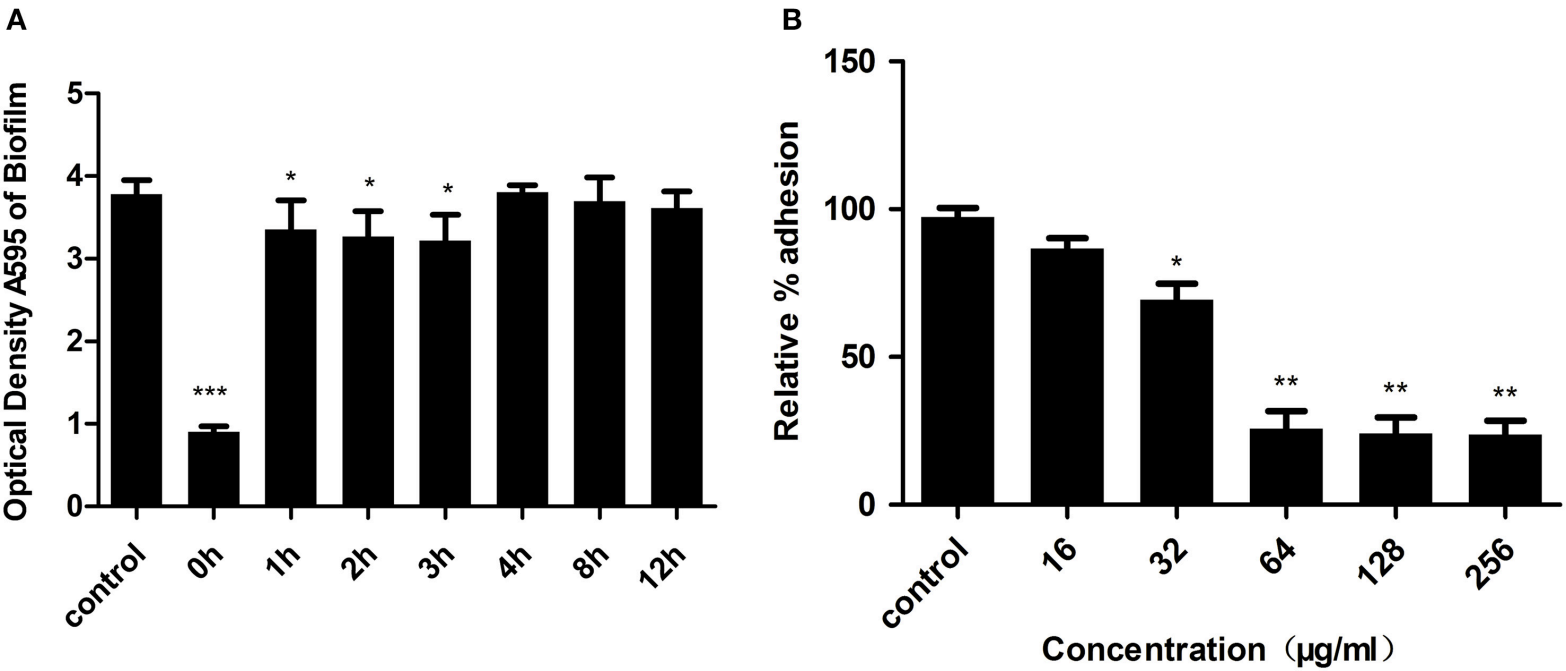
**(A)** Kaempferol specifically inhibited the attachment of phase biofilm formation. Kaempferol was added at various time points during biofilm formation, and the effects were measured after a total of 20 h of incubation. CV was used to stain the biofilms. The bound CV was released from the stained cells with 95% ethanol, and the OD595 was measured. Data are represented as the mean ± standard deviation. ^*^P < 0.05, and ^***^P < 0.001 compared to the control. **(B)** Relative % adhesion of *S. aureus* to fibrinogen. Bacteria treated with kaempferol were cultured in 96-well plates coated with 20 μg/ml fibrinogen at 37°C for 1 h. The OD595 was measured as described above. The relative % adhesion was calculated. ^**^P < 0.05, and ^**^P < 0.01.

The development of bacterial biofilm can be divided into the following three phases, which involve specific molecular factors: attachment, maturation, and detachment (Otto, 2013). To explore which phases of biofilm development kaempferol influenced, we added kaempferol (64 μg/ml) at different time points during biofilm formation and measured the effects after a total of 20 h of incubation. There was no inhibitor in the control group. As shown in Figure 2A, compared to the control group, kaempferol significantly inhibited the formation of biofilms only when added immediately after inoculation (0 h). After bacteria were incubated for 1, 2, 3, 4, 8, and 12 h, the addition of kaempferol had no effect and the biofilms were completely resistant to kaempferol. These results demonstrated that kaempferol specifically inhibited the attachment phase of biofilm formation.

The above results showed that kaempferol only affected the attachment phase of biofilm formation, and this phase was primarily mediated by the binding of *S. aureus* surface-anchored proteins and host matrix proteins (Otto, 2013). Fibrinogen, as a plasma protein, can be used as a substrate for staphylococcal adhesion (Patti et al., 1994). To examine the influence of kaempferol on *S. aureus* adhesion, we therefore employed a fibrinogen-bing assay in which cell adhesion to fibrinogen-coated plates was stained with crystal violet and quantified by measuring the absorbance at 595 nm. The relative % adhesion was reduced after treatment with kaempferol at 32 μg/ml compared to the control group. When treating with 64 μg/ml of kaempferol, the relative % adhesion decreased by approximately 75% (Figure 2B). These results indicated that kaempferol inhibited the formation of biofilm by reducing *S. aureus* adhesion.

### Kaempferol prevents the formation of *S. aureus* biofilms by inhibiting the activity of sortase A

**Figure 3:**
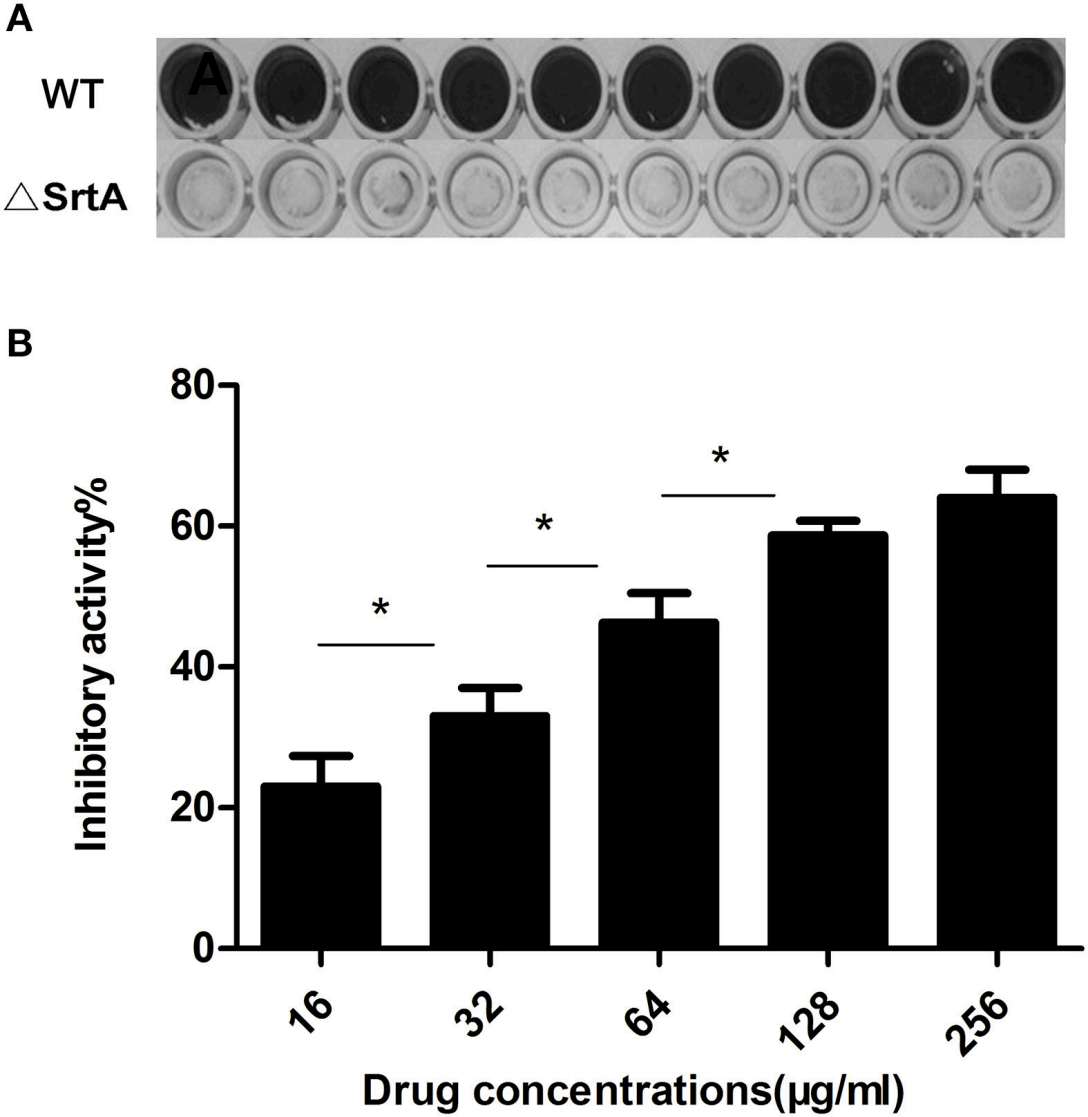
**(A)** Wild-type and ΔSrtA *S. aureus* biofilms. **(B)** Inhibitory effect of kaempferol on the activity of *S. aureus* SrtA *in Vitro*. ^*^P < 0.05.

*S. aureus* surface proteins include clumping factors (ClfA and ClfB), which are essential for the adhesion of *S. aureus* to fibrinogen (Bi et al., 2016). These proteins are mainly anchored by sortase A to the cell wall and play significant roles in the formation of biofilms (Cascioferro et al., 2014). Thus, we cultured the biofilm of a sortase A-null mutant of *S. aureus* (ΔSrtA). The result shown in Figure 3A proved that the ΔSrtA strain had no ability to form biofilms, further indicating that SrtA-mediated surface proteins were essential for this strain (*S. aureus* ATCC® 29213™) to form biofilms under these conditions. Based on this result, to test the effect of kaempferol on the activity of SrtA, we performed a FRET (Ton-That et al., 1999; Mazmanian et al., 2002) assay *in vitro* in which a fluorescent peptide substrate Dabcyl-QALPETGEE-Edans of SrtA was used to monitor the fluorescence changes during SrtA catalysis. The result showed that SrtA activity was blocked by 47% (Figure 3B) after treatment with 64 μg/ml kaempferol, suggesting that kaempferol weakened the adhesion of *S. aureus* by blocking the activity of SrtA, resulting in the inhibition of biofilm formation.

**Figure 4:**
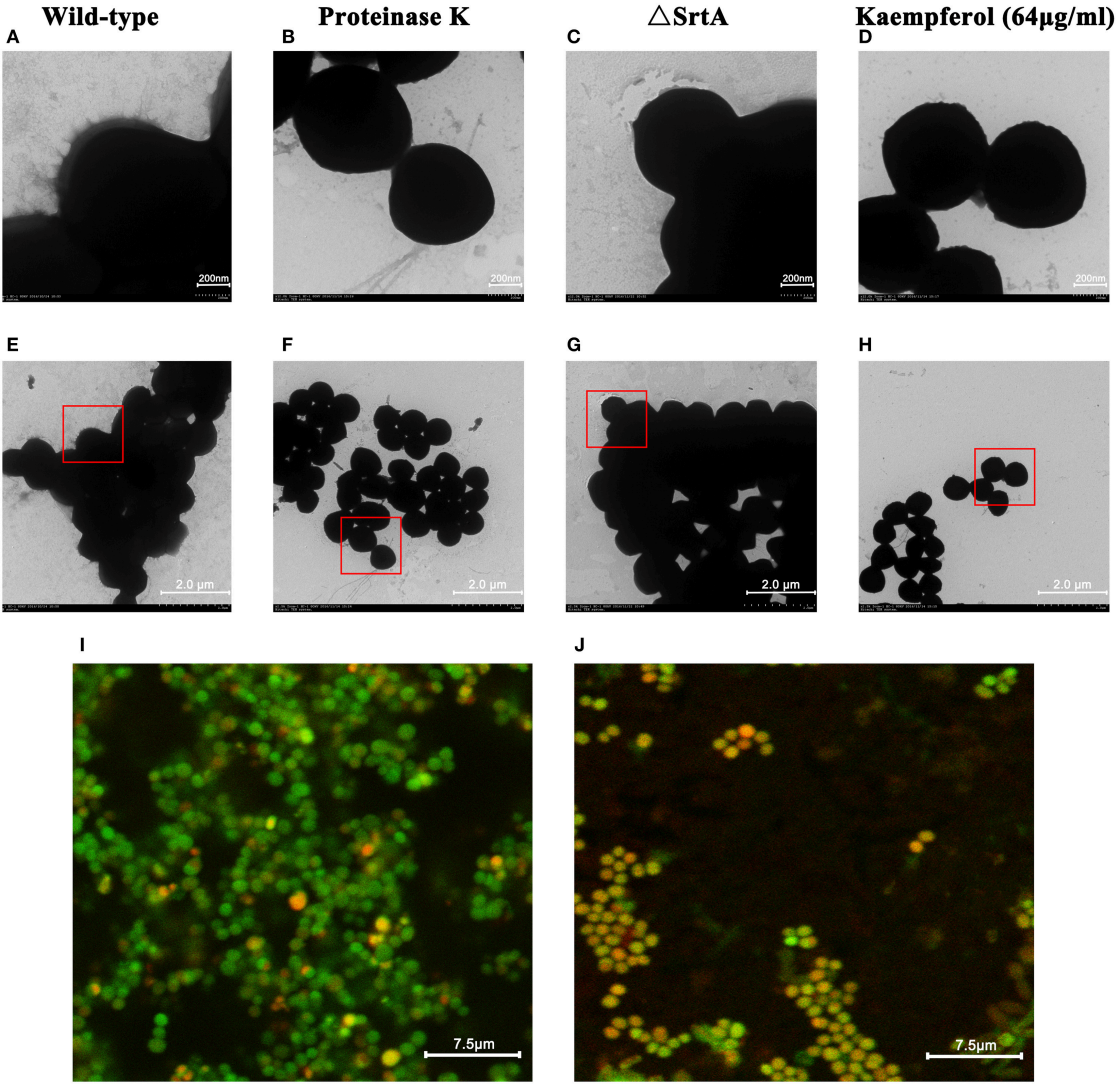
TEM. **(A,E)** Wild-type group. **(B,F)** Proteinase K group. **(C,G)** ΔSrtA group. **(D,H)** Kaempferol 64 μg/ml group. Confocal microscopy images of *S. aureus* biofilms grown with or without kaempferol. **(I)** Wild-type group. **(J)** Kaempferol 64 μg/ml group.

In addition, under a transmission electron microscope (TEM), we observed many fibrous protrusions around the wild-type strain (Figures 4A,E). Biofilms treated with proteinase K were dispersed (data not shown) and the fibrous protrusions disappeared (Figures 4B,F). As shown in Figures 4C,G, the surface of ΔSrtA strain was also smooth. A significant reduction of fibrous protrusions on the surface of bacteria was observed after treatment with 64 μg/ml kaempferol (Figures 4D,H). Moreover, we chose two fluorescent dyes, Syto63 and FITC. The intracellular DNA were stained red with Syto63 dye and the extracellular proteins were stained green with FITC dye. Under confocal laser microscopy, there was obvious green fluorescence around the wild-type strain (Figure 4I) and the green fluorescence decreased after treatment with kaempferol at 64 μg/ml (Figure 4J).

Based on these data, we inferred that kaempferol reduced the anchoring of surface proteins by inhibiting the activity of SrtA, which led to biofilm formation inhibited.

### Kaempferol modulates the expression of *S. aureus* adhesion-related genes

**Figure 5:**
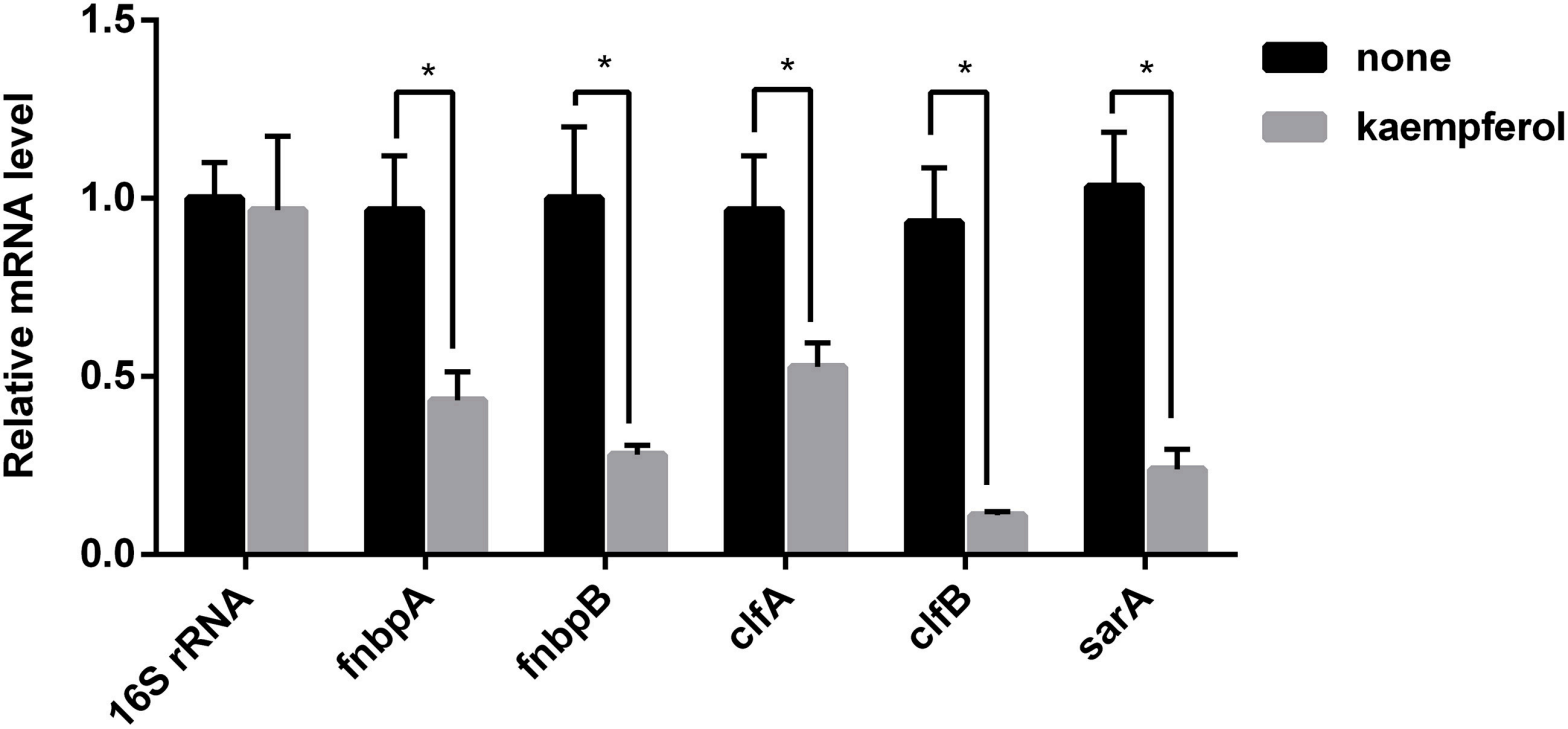
Transcription profiles of *S. aureus* cells treated with or without kaempferol. *S. aureus* ATCC® 29213™ was cultivated to an A600 of 1 and incubated with or without kaempferol (64 μg/ml) for 5 h with shaking at 220 rpm. Transcriptional profiles were measured by qRT-PCR. The expression level of 16S rRNA was used to normalize the expression of the genes of interest. The experiment was performed in triplicate (3 qRT-PCR replicates were performed per gene). ^*^P < 0.05 vs. non-treated controls (none).

According to the preceding conclusions, kaempferol blocked the activity of SrtA by only 47% at 64 μg/ml, whereas inhibited the biofilm formation of *S. aureus* by 80% at this concentration. So, there may be other targets for kaempferol to affect the biofilm formation. Above results illustrated that kaempferol only acted on the adhesion stage of biofilm formation. To further investigate the molecular mechanism of adhesion inhibition, qRT-PCR was conducted to detect the transcription level of several adhesion-related genes. As shown in Figure 5, the expression of several genes was altered. The genes *clfA* and *clfB*, which encode Clumping factor A (ClfA) and ClfB were repressed by 45 and 88%. In addition, kaempferol also down-regulated *fnbA* and *fnbB* which encode Fibronectin-binding proteins (FnbpA and FnbpB). The inhibition rates were 56 and 72%. The global regulatory gene *sarA* was studied here as well and it was inhibited by 77%. The expression level of the 16S rRNA gene served as the internal control. These results suggested that kaempferol down-regulated the expression of adhesion-related genes, which was responsible to explain its inhibitory effect on the biofilm formation.

## Discussion

The increasing rate of antibiotic resistance makes *S. aureus* a major cause of hospital infections. In *S. aureus*, biofilm formation is a mechanism of antibiotic resistance, further limiting the efficacy of antibiotics by creating a physical barrier and due to differences in metabolism (Hochbaum et al., 2011). The appearance of multidrug-resistant *S. aureus* makes it urgent to find a more effective way to treat biofilm-associated infections.

During the initial adhesion phase, cell wall-anchored (CWA) proteins of *S. aureus* play an important role in adhesion to biotic surfaces surrounded by host tissues and to abiotic surfaces coated with plasma proteins. Bacterial surface proteins that bind human matrix proteins are collectively known as microbial surface components recognizing adhesive matrix molecules (MSCRAMMs) (Otto, 2014). Some MSCRAMMs are known to be related to the early stages of biofilm formation, such as FnBPA, FnBPB (Massey et al., 2002; Foster et al., 2014), and the clumping factors ClfA (McDevitt et al., 1997) and ClfB (Ní Eidhin et al., 1998). Most MSCRAMMs in *S. aureus* have a common cell wall-targeting motif (LPXTG) and are targeted to bacterial surfaces via sortase A (SrtA), which catalyzes the covalent attachment of these proteins to the penta-glycine cross-linker component of the peptidoglycan (Mazmanian et al., 1999) and plays significant roles in bacterial adhesion (Cascioferro et al., 2014; Zapotoczna et al., 2015).

Our results showed that kaempferol reduced the adhesion of bacteria to fibrinogen, which is the first step in the formation of *S. aureus* biofilms (Götz, 2002). One possible mechanism of action is that kaempferol destroys the activity of SrtA and thus hinders the anchoring of surface proteins. FRET (Ton-That et al., 1999; Mazmanian et al., 2002) was used to examine the inhibitory activity of kaempferol against SrtA *in vitro*. An enzyme activity assay indicated that the activity of SrtA was reduced by approximately 47% at 64 μg/ml (Figure 3B), whereas the biofilm was decreased by 80% at this concentration. This result suggests that the inhibition of biofilm formation is not absolutely dependent on the inhibition of the SrtA activity.

Another possible mechanism is that kaempferol represses the gene expression of some surface proteins. Since kaempferol specifically affects the attachment phase of biofilm formation, we selected genes associated with adhesion protein expression. The clumping factors ClfA and ClfB encoded by the genes *clfA* and *clfB* are the most important proteins for the binding of *S. aureus* to fibrinogen (Gowrishankar et al., 2016). In *S. aureus*, ClfA and ClfB are fibrinogen-binding proteins (Otto, 2004; Foster et al., 2014) that are up-regulated during the biofilm growth (Resch et al., 2005). In addition to fibrinogen-binding proteins, *S. aureus* has two fibronectin-binding proteins, FnBPA and FnBPB, which are encoded by *fnbA* and *fnbB*, respectively (Jonsson et al., 1991). FnBPs are thought to promote biofilm formation using a self-association mechanism distinct from ligand binding, making them multifunctional in the *S. aureus* biofilm life cycle (Geoghegan et al., 2013; Herman-Bausier et al., 2015).

In *S. aureus*, SarA is a global regulator that is closely related to biofilm formation *in vitro* (Paharik and Horswill, 2016). Thus, the global regulatory gene *sarA* was examined in this study. The effect of kaempferol on the transcription level of adhesion-related genes was tested by qRT-PCR. As shown in Figure 5, the transcription levels of *clfA, clfB, fnbB, fnbA, and sarA* were reduced by kaempferol. SarA is required to form biofilm in *S. aureus* (Beenken et al., 2003; Zielinska et al., 2012) and can positively regulate *fnbA* and *fnbB* (Dunman et al., 2001). Moreover, the inactivation of SarA resulted in decreased production of fibronectin-binding protein and fibrinogen-binding protein (Arvidson and Tegmark, 2001). We infer from these results that kaempferol is likely to act on the expression of these genes and lead to the biofilm formation inhibited.

In addition, by TEM, we observed many fibrous protrusions around the wild-type strain (Figures 4A,E). After treatment with proteinase K, the biofilm dispersed, and the fibrous protrusions disappeared (Figures 4B,F). Thus, we speculated that the fibrous protrusions were important for biofilm formation and consisted mainly of proteins. Moreover, by confocal laser microscopy, we observed significant green fluorescence around the wild-type strain (Figure 4I) because of the proteins surrounding the bacteria. We observed a reduction in green fluorescence (Figure 4J) after treatment with kaempferol (64 μg/ml). According to this result, we further inferred that the fibrous protrusions mainly consisted of proteins and that kaempferol could reduce the production of these proteins. Moreover, the surface of the ΔSrtA strain is also smooth (Figures 4C,G), and this strain has no ability to form biofilm (Figure 3A), indicating that surface proteins mediated by SrtA may be the major components of the fibrous protrusions and are important for biofilm formation. We observed a significant reduction in fibrous protrusions on the surface of bacteria treated with 64 μg/ml kaempferol (Figures 4D,H). According to these results, we conclude that kaempferol inhibits biofilm formation by reducing *S. aureus* surface proteins.

In a word, kaempferol can prevent *S. aureus* biofilm formation effectively even at sub-inhibitory concentrations. It is possible to be used on medical devices as anti-biofilm coatings to prevent infections. Therefore, we believe that kaempferol is a potential compound with a novel mechanism of biofilm inhibition, which could provide a lead structure for the development of future anti-biofilm drugs.

## Author contributions

Conceived and designed the experiments: DMi, DW, FC, LW, and TW. Performed the experiments: DMi, FC, DMu, JC, BL, and LZ. Analyzed the data: DMi, FC, LW, HX, XD and XZ. Wrote the paper: DMi, DW, and LW. All authors participated in discussion about the results and the manuscript.

### Conflict of interest statement

The authors declare that the research was conducted in the absence of any commercial or financial relationships that could be construed as a potential conflict of interest.

## References

[B1] Aaron S. D. Ferris W. Ramotar K. Vandemheen K. Chan F. Saginur R. (2002). Single and combination antibiotic susceptibilities of planktonic, adherent, and biofilm-grown *Pseudomonas aeruginosa* isolates cultured from sputa of adults with cystic fibrosis. J. Clin. Microbiol. 40, 4172–4179. 10.1128/JCM.40.11.4172-4179.2002 12409393 PMC139693

[B2] Abouelhassan Y. Garrison A. T. Burch G. M. Wong W. Norwood V. M. IV. Huigens R. W. III. (2014). Discovery of quinoline small molecules with potent dispersal activity against methicillin-resistant *Staphylococcus aureus* and *Staphylococcus epidermidis* biofilms using a scaffold hopping strategy. Bioorg. Med. Chem. Lett. 24, 5076–5080. 10.1016/j.bmcl.2014.09.009 25264073

[B3] Aendekerk S. Diggle S. P. Song Z. Hoiby N. Cornelis P. Williams P.. (2005). The MexGHI-OpmD multidrug efflux pump controls growth, antibiotic susceptibility and virulence in *Pseudomonas aeruginosa* via 4-quinolone-dependent cell-to-cell communication. Microbiology 151, 1113–1125. 10.1099/mic.0.27631-0 15817779

[B4] Anderson G. G. O'Toole G. A. (2008). Innate and induced resistance mechanisms of bacterial biofilms, in Bacterial Biofilms, ed Romeo T. (Berlin; Heidelberg: Springer), 85–105. 10.1007/978-3-540-75418-3_5 18453273

[B5] Arvidson S. Tegmark K. (2001). Regulation of virulence determinants in *Staphylococcus aureus*. Int. J. Med. Microbiol. 291, 159–170. 10.1078/1438-4221-00112 11437338

[B6] Beenken K. E. Blevins J. S. Smeltzer M. S. (2003). Mutation of sarA in *Staphylococcus aureus* limits biofilm formation. Infect. Immun. 71, 4206–4211. 10.1128/IAI.71.7.4206-4211.2003 12819120 PMC161964

[B7] Bi C. Dong X. Zhong X. Cai H. Wang D. Wang L. (2016). Acacetin protects mice from *Staphylococcus aureus* bloodstream infection by inhibiting the activity of sortase A. Molecules 21:E1285. 10.3390/molecules21101285 27681715 PMC6272931

[B8] Bjarnsholt T. Jensen P. O. Burmolle M. Hentzer M. Haagensen J. A. J. Hougen H. P.. (2005). *Pseudomonas aeruginosa* tolerance to tobramycin, hydrogen peroxide and polymorphonuclear leukocytes is quorum-sensing dependent. Microbiology 151, 373–383. 10.1099/mic.0.27463-0 15699188

[B9] Boles B. R. Horswill A. R. (2011). Staphylococcal biofilm disassembly. Trends Microbiol. 19, 449–455. 10.1016/j.tim.2011.06.004 21784640 PMC3164736

[B10] Brackman G. Coenye T. (2015). Quorum sensing inhibitors as anti-biofilm agents. Curr. Pharm. Des. 21, 5–11. 10.2174/1381612820666140905114627 25189863

[B11] Brown A. R. Ettefagh K. A. Todd D. Cole P. S. Egan J. M. Foil D. H.. (2015). A mass spectrometry-based assay for improved quantitative measurements of efflux pump inhibition. PLoS ONE 10:e0124814. 10.1371/journal.pone.0124814 25961825 PMC4427306

[B12] Cascioferro S. Totsika M. Schillaci D. (2014). Sortase A: an ideal target for anti-virulence drug development. Microb. Pathog. 77, 105–112. 10.1016/j.micpath.2014.10.007 25457798

[B13] Chen F. Liu B. Wang D. Wang L. Deng X. Bi C.. (2014). Role of sortase a in the pathogenesis of *Staphylococcus aureus*-induced mastitis in mice. FEMS Microbiol. Lett. 351, 95–103. 10.1111/1574-6968.12354 24330077

[B14] Cho H. S. Lee J.-H. Cho M. H. Lee J. (2015). Red wines and flavonoids diminish *Staphylococcus aureus* virulence with anti-biofilm and anti-hemolytic activities. Biofouling 31, 1–11. 10.1080/08927014.2014.991319 25535776

[B15] Chu M. Zhang M.-B. Liu Y.-C. Kang J.-R. Chu Z.-Y. Yin K.-L.. (2016). Role of berberine in the treatment of methicillin-resistant *Staphylococcus aureus* infections. Sci. Rep. 6:24748. 10.1038/srep24748 27103062 PMC4840435

[B16] Chung P. Y. Toh Y. S. (2014). Anti-biofilm agents: recent breakthrough against multi-drug resistant *Staphylococcus aureus*. Pathog. Dis. 70, 231–239. 10.1111/2049-632X.12141 24453168

[B17] Costerton J. W. Stewart P. S. Greenberg E. P. (1999). Bacterial biofilms: a common cause of persistent infections. Science 284, 1318–1322. 10.1126/science.284.5418.1318 10334980

[B18] Dunman P. M. Murphy E. Haney S. Palacios D. Tucker-Kellogg G. Wu S. (2001). Transcription profiling-based identification of *Staphylococcus aureus* genes regulated by the agr and/or sarA loci. J. Bacteriol. 183, 7341–7353. 10.1128/JB.183.24.7341-7353.2001 11717293 PMC95583

[B19] Foster T. J. Geoghegan J. A. Ganesh V. K. Hook M. (2014). Adhesion, invasion and evasion: the many functions of the surface proteins of *Staphylococcus aureus*. Nat. Rev. Microbiol. 12, 49–62. 10.1038/nrmicro3161 24336184 PMC5708296

[B20] Geoghegan J. A. Monk I. R. O'Gara J. P. Foster T. J. (2013). Subdomains N2N3 of fibronectin binding protein a mediate *Staphylococcus aureus* biofilm formation and adherence to fibrinogen using distinct mechanisms. J. Bacteriol. 195, 2675–2683. 10.1128/JB.02128-12 23564165 PMC3676058

[B21] Götz F. (2002). *Staphylococcus* and biofilms. Mol. Microbiol. 43, 1367–1378. 10.1046/j.1365-2958.2002.02827.x 11952892

[B22] Gowrishankar S. Kamaladevi A. Balamurugan K. Pandian S. K. (2016). *In vitro* and *in vivo* biofilm characterization of methicillin-resistant staphylococcus aureus from patients associated with pharyngitis infection. Biomed Res. Int. 2016:1289157. 10.1155/2016/1289157 27761465 PMC5059529

[B23] Harmsen M. Lappann M. Knøchel S. Molin S. (2010). Role of extracellular DNA during biofilm formation by listeria monocytogenes. Appl. Environ. Microbiol. 76, 2271–2279. 10.1128/AEM.02361-09 20139319 PMC2849236

[B24] Herman-Bausier P. El-Kirat-Chatel S. Foster T. J. Geoghegan J. A. Dufrene Y. F. (2015). *Staphylococcus aureus* fibronectin-binding protein a mediates cell-cell adhesion through low-affinity homophilic bonds. mBio 6:e00413–15. 10.1128/mBio.00413-15 26015495 PMC4447249

[B25] Hochbaum A. I. Kolodkin-Gal I. Foulston L. Kolter R. Aizenberg J. Losick R. (2011). Inhibitory effects of D-amino acids on *Staphylococcus aureus* biofilm development. J. Bacteriol. 193, 5616–5622. 10.1128/JB.05534-11 21856845 PMC3187230

[B26] Hoyle B. D. Costerton J. W. (1991). Bacterial resistance to antibiotics: the role of biofilms, in Progress in Drug Research/Fortschritte der Arzneimittelforschung/Progrès des Recherches Pharmaceutiques, ed Jucker E. (Basel: Birkhäuser Basel), 91–105. 10.1007/978-3-0348-7139-6_2 1763187

[B27] Huang Y. H. Huang C. C. Chen C. C. Yang K. J. Huang C. Y. (2015). Inhibition of *Staphylococcus aureus* PriA helicase by flavonol kaempferol. Protein J. 34, 169–172. 10.1007/s10930-015-9609-y 25894858 PMC7088215

[B28] Itoh Y. Wang X. Hinnebusch B. J. Preston J. F. III. Romeo T. (2005). Depolymerization of β-1,6-N-acetyl-D-glucosamine disrupts the integrity of diverse bacterial biofilms. J. Bacteriol. 187, 382–387. 10.1128/JB.187.1.382-387.2005 15601723 PMC538831

[B29] Jia M. Chen Z. Guo Y. Chen X. Zhao X. (2017). Efficacy of silk fibroin-nano silver against *Staphylococcus aureus* biofilms in a rabbit model of sinusitis. Int. J. Nanomed. 12, 2933–2939. 10.2147/IJN.S130160 28435269 PMC5391841

[B30] Jonsson K. Signas C. Muller H. P. Lindberg M. (1991). Two different genes encode fibronectin binding proteins in *Staphylococcus aureus*. The complete nucleotide sequence and characterization of the second gene. Eur. J. Biochem. 202, 1041–1048. 10.1111/j.1432-1033.1991.tb16468.x 1837266

[B31] Kelly D. McAuliffe O. Ross R. P. Coffey A. (2012). Prevention of *Staphylococcus aureus* biofilm formation and reduction in established biofilm density using a combination of phage K and modified derivatives. Lett. Appl. Microbiol. 54, 286–291. 10.1111/j.1472-765X.2012.03205.x 22251270

[B32] Lee J.-H. Kim Y.-G. Ryu S. Y. Cho M. H. Lee J. (2014). Ginkgolic acids and Ginkgo biloba extract inhibit *Escherichia coli* O157:H7 and *Staphylococcus aureus* biofilm formation. Int. J. Food Microbiol. 174, 47–55. 10.1016/j.ijfoodmicro.2013.12.030 24457153

[B33] Lee J.-H. Kim Y.-G. Yong Ryu S. Lee J. (2016). Calcium-chelating alizarin and other anthraquinones inhibit biofilm formation and the hemolytic activity of *Staphylococcus aureus*. Sci. Rep. 6:19267. 10.1038/srep19267 26763935 PMC4725881

[B34] Lee J.-H. Park J.-H. Cho H. S. Joo S. W. Cho M. H. Lee J. (2013). Anti-biofilm activities of quercetin and tannic acid against *Staphylococcus aureus*. Biofouling 29, 491–499. 10.1080/08927014.2013.788692 23668380

[B35] Li J. W.-H. Vederas J. C. (2009). Drug discovery and natural products: end of an era or an endless frontier? Science 325, 161–165. 10.1126/science.1168243 19589993

[B36] Livak K. J. Schmittgen T. D. (2001). Analysis of relative gene expression data using real-time quantitative PCR and the 2^−ΔΔC^T method. Methods 25, 402–408. 10.1006/meth.2001.1262 11846609

[B37] Lowy F. D. (1998). *Staphylococcus aureus* infections. N. Engl. J. Med. 339, 520–532. 10.1056/NEJM199808203390806 9709046

[B38] Mah T. F. O'Toole G. A. (2001). Mechanisms of biofilm resistance to antimicrobial agents. Trends Microbiol. 9, 34–39. 10.1016/S0966-842X(00)01913-2 11166241

[B39] Massey R. C. Dissanayeke S. R. Cameron B. Ferguson D. Foster T. J. Peacock S. J. (2002). Functional blocking of *Staphylococcus aureus* adhesins following growth in *ex vivo* media. Infect. Immun. 70, 5339–5345. 10.1128/IAI.70.10.5339-5345.2002 12228257 PMC128300

[B40] Mazmanian S. K. Liu G. Ton-That H. Schneewind O. (1999). *Staphylococcus aureus* sortase, an enzyme that anchors surface proteins to the cell wall. Science 285, 760–763. 10.1126/science.285.5428.760 10427003

[B41] Mazmanian S. K. Ton-That H. Su K. Schneewind O. (2002). An iron-regulated sortase anchors a class of surface protein during *Staphylococcus aureus* pathogenesis. Proc. Natl. Acad. Sci. U.S.A. 99, 2293–2298. 10.1073/pnas.032523999 11830639 PMC122358

[B42] McDevitt D. Nanavaty T. House-Pompeo K. Bell E. Turner N. McIntire L.. (1997). Characterization of the interaction between the *Staphylococcus aureus* clumping factor (ClfA) and fibrinogen. Eur. J. Biochem. 247, 416–424. 10.1111/j.1432-1033.1997.00416.x 9249055

[B43] Mistry H. Sharma P. Mahato S. Saravanan R. Kumar P. A. Bhandari V. (2016). Prevalence and characterization of oxacillin susceptible meca-positive clinical isolates of staphylococcus aureus causing bovine mastitis in India. PLoS ONE 11:e0162256. 10.1371/journal.pone.0162256 27603123 PMC5014444

[B44] Mogosanu G. D. Grumezescu A. M. Huang K.-S. Bejenaru L. E. Bejenaru C. (2015). Prevention of microbial communities: novel approaches based natural products. Curr. Pharm. Biotechnol. 16, 94–111. 10.2174/138920101602150112145916 25594287

[B45] Ní Eidhin D. Perkins S. Francois P. Vaudaux P. Höök M. Foster T. J. (1998). Clumping factor B (ClfB), a new surface-located fibrinogen-binding adhesin of *Staphylococcus aureus*. Mol. Microbiol. 30, 245–257. 10.1046/j.1365-2958.1998.01050.x 9791170

[B46] O'Gara J. P. Humphreys H. (2001). Staphylococcus epidermidis biofilms: importance and implications. J. Med. Microbiol. 50, 582–587. 10.1099/0022-1317-50-7-582 11444767

[B47] Otto M. (2004). Virulence factors of the coagulase-negative staphylococci. Front. Biosci. 9, 841–863. 10.2741/1295 14766414

[B48] Otto M. (2008). Staphylococcal biofilms. Curr. Top. Microbiol. Immunol. 322, 207–228. 10.1007/978-3-540-75418-3_10 18453278 PMC2777538

[B49] Otto M. (2013). Staphylococcal infections: mechanisms of biofilm maturation and detachment as critical determinants of pathogenicity. Annu. Rev. Med. 64, 175–188. 10.1146/annurev-med-042711-140023 22906361

[B50] Otto M. (2014). Physical stress and bacterial colonization. FEMS Microbiol. Rev. 38, 1250–1270. 10.1111/1574-6976.12088 25212723 PMC4227950

[B51] Paharik A. E. Horswill A. R. (2016). The staphylococcal biofilm: adhesins, regulation, and host response. Microbiol. Spectr. 4, 1–27. 10.1128/microbiolspec.VMBF-0022-2015 27227309 PMC4887152

[B52] Parsek M. R. Singh P. K. (2003). Bacterial biofilms: an emerging link to disease pathogenesis. Annu. Rev. Microbiol. 57, 677–701. 10.1146/annurev.micro.57.030502.090720 14527295

[B53] Patti J. M. Allen B. L. McGavin M. J. Hook M. (1994). MSCRAMM-mediated adherence of microorganisms to host tissues. Annu. Rev. Microbiol. 48, 585–617. 10.1146/annurev.mi.48.100194.003101 7826020

[B54] Payne D. E. Martin N. R. Parzych K. R. Rickard A. H. Underwood A. Boles B. R. (2013). Tannic acid inhibits *Staphylococcus aureus* surface colonization in an IsaA-dependent manner. Infect. Immun. 81, 496–504. 10.1128/IAI.00877-12 23208606 PMC3553799

[B55] Pletzer D. Hancock R. E. W. (2016). Antibiofilm peptides: potential as broad-spectrum agents. J. Bacteriol. 198, 2572–2578. 10.1128/JB.00017-16 27068589 PMC5019066

[B56] Qiu J. Niu X. Dong J. Wang D. Wang J. Li H.. (2012). Baicalin protects mice from *Staphylococcus aureus* pneumonia via inhibition of the cytolytic activity of alpha-hemolysin. J. Infect. Dis. 206, 292–301. 10.1093/infdis/jis336 22551812

[B57] Quave C. L. Estevez-Carmona M. Compadre C. M. Hobby G. Hendrickson H. Beenken K. E.. (2012). Ellagic acid derivatives from *Rubus ulmifolius* inhibit *Staphylococcus aureus* biofilm formation and improve response to antibiotics. PLoS ONE 7:e28737. 10.1371/journal.pone.0028737 22242149 PMC3252291

[B58] Rendueles O. Kaplan J. B. Ghigo J.-M. (2013). Antibiofilm polysaccharides. Environ. Microbiol. 15, 334–346. 10.1111/j.1462-2920.2012.02810.x 22730907 PMC3502681

[B59] Resch A. Rosenstein R. Nerz C. Gotz F. (2005). Differential gene expression profiling of *Staphylococcus aureus* cultivated under biofilm and planktonic conditions. Appl. Environ. Microbiol. 71, 2663–2676. 10.1128/AEM.71.5.2663-2676.2005 15870358 PMC1087559

[B60] Rogers S. A. Huigens R. W. III. Cavanagh J. Melander C. (2010). Synergistic effects between conventional antibiotics and 2-aminoimidazole-derived antibiofilm agents. Antimicrob. Agents Chemother. 54, 2112–2118. 10.1128/AAC.01418-09 20211901 PMC2863642

[B61] Romling U. Balsalobre C. (2012). Biofilm infections, their resilience to therapy and innovative treatment strategies. J. Intern. Med. 272, 541–561. 10.1111/joim.12004 23025745

[B62] Ross J. A. Kasum C. M. (2002). Dietary flavonoids: bioavailability, metabolic effects, and safety. Annu. Rev. Nutr. 22, 19–34. 10.1146/annurev.nutr.22.111401.144957 12055336

[B63] Singhal D. Foreman A. Bardy J. J. Wormald P. J. (2011). *Staphylococcus aureus* biofilms: nemesis of endoscopic sinus surgery. Laryngoscope 121, 1578–1583. 10.1002/lary.21805 21647904

[B64] Ton-That H. Liu G. Mazmanian S. K. Faull K. F. Schneewind O. (1999). Purification and characterization of sortase, the transpeptidase that cleaves surface proteins of *Staphylococcus aureus* at the LPXTG motif. Proc. Natl. Acad. Sci. U.S.A. 96, 12424–12429. 10.1073/pnas.96.22.12424 10535938 PMC22937

[B65] Von Eiff C. Peters G. Heilmann C. (2002). Pathogenesis of infections due to coagulase-negative staphylococci. Lancet Infect. Dis. 2, 677–685. 10.1016/S1473-3099(02)00438-3 12409048

[B66] Wang D. Jin Q. Xiang H. Wang W. Guo N. Zhang K.. (2011). Transcriptional and functional analysis of the effects of magnolol: inhibition of autolysis and biofilms in *Staphylococcus aureus*. PLoS ONE 6:e26833. 10.1371/journal.pone.0026833 22046374 PMC3203910

[B67] Wang J. Qiu J. Tan W. Zhang Y. Wang H. Zhou X.. (2015). Fisetin inhibits *Listeria* monocytogenes virulence by interfering with the oligomerization of listeriolysin O. J. Infect. Dis. 211, 1376–1387. 10.1093/infdis/jiu520 25231018

[B68] Wang L. Bi C. Cai H. Liu B. Zhong X. Deng X.. (2015). The therapeutic effect of chlorogenic acid against *Staphylococcus aureus* infection through sortase a inhibition. Front. Microbiol. 6:1031. 10.3389/fmicb.2015.01031 26528244 PMC4608362

[B69] Yadav M. K. Chae S.-W. Im G. J. Chung J.-W. Song J.-J. (2015). Eugenol: a phyto-compound effective against methicillin-resistant and methicillin-sensitive *Staphylococcus aureus* clinical strain biofilms. PLoS ONE 10:e0119564. 10.1371/journal.pone.0119564 25781975 PMC4364371

[B70] Zapotoczna M. McCarthy H. Rudkin J. K. O'Gara J. P. O'Neill E. (2015). An essential role for coagulase in *Staphylococcus aureus* biofilm development reveals new therapeutic possibilities for device-related infections. J. Infect. Dis. 212, 1883–1893. 10.1093/infdis/jiv319 26044292

[B71] Zielinska A. K. Beenken K. E. Mrak L. N. Spencer H. J. Post G. R. Skinner R. A.. (2012). sarA-mediated repression of protease production plays a key role in the pathogenesis of *Staphylococcus aureus* USA300 isolates. Mol. Microbiol. 86, 1183–1196. 10.1111/mmi.12048 23075270 PMC3508076

